# Rectification of the Water Permeability in COS-7 Cells at 22, 10 and 0°C

**DOI:** 10.1371/journal.pone.0023643

**Published:** 2011-08-24

**Authors:** Diana B. Peckys, F. W. Kleinhans, Peter Mazur

**Affiliations:** 1 Department of Molecular Physiology and Biophysics, Vanderbilt University School of Medicine, Nashville, Tennessee, United States of America; 2 Department of Physics, Indiana University-Purdue University at Indianapolis, Indianapolis, Indiana, United States of America; 3 Department of Biochemistry, and Cellular and Molecular Biology, University of Tennessee, Knoxville, Tennessee, United States of America; University of California at Berkeley, United States of America

## Abstract

The osmotic and permeability parameters of a cell membrane are essential physico-chemical properties of a cell and particularly important with respect to cell volume changes and the regulation thereof. Here, we report the hydraulic conductivity, L_p_, the non-osmotic volume, V_b_, and the Arrhenius activation energy, E_a_, of mammalian COS-7 cells. The ratio of V_b_ to the isotonic cell volume, V_c iso_, was 0.29. E_a_, the activation energy required for the permeation of water through the cell membrane, was 10,700, and 12,000 cal/mol under hyper- and hypotonic conditions, respectively. Average values for L_p_ were calculated from swell/shrink curves by using an integrated equation for L_p_. The curves represented the volume changes of 358 individually measured cells, placed into solutions of nonpermeating solutes of 157 or 602 mOsm/kg (at 0, 10 or 22°C) and imaged over time. L_p_ estimates for all six combinations of osmolality and temperature were calculated, resulting in values of 0.11, 0.21, and 0.10 µm/min/atm for exosmotic flow and 0.79, 1.73 and 1.87 µm/min/atm for endosmotic flow (at 0, 10 and 22°C, respectively). The unexpected finding of several fold higher L_p_ values for endosmotic flow indicates highly asymmetric membrane permeability for water in COS-7. This phenomenon is known as rectification and has mainly been reported for plant cell, but only rarely for animal cells. Although the mechanism underlying the strong rectification found in COS-7 cells is yet unknown, it is a phenomenon of biological interest and has important practical consequences, for instance, in the development of optimal cryopreservation.

## Introduction

Water transport across cellular membranes is of crucial importance in animal and plant physiology. The permeability of a cell to water and the temperature coefficient of that permeability are two of its more important parameters. They, along with a cell's permeability or lack thereof to solutes, determine the magnitude and kinetics of cell volume changes when the cell is subjected to conditions that depart from isotonic or isoosmotic. The permeability of a cell to water is usually referred to as the hydraulic conductivity, L_p_. It has the units of volume divided by area×pressure×time, or commonly, µm/atm. min.

Water permeability is of particular importance in cryobiology, which is the main focus in our laboratory. The value of L_p_ is one of the chief factors determining the conditions under which ice forms or does not form in the interior of a cell [1], [2]. Intracellular ice formation (IIF) is almost always lethal. If the cooling rate is low enough or if the L_p_ is high enough, the cell will dehydrate during cooling and will not undergo IIF. In contrast, if the cooling rate is too high or the L_p_ too low, the cell will not dehydrate rapidly enough to maintain osmotic or chemical equilibrium with the external ice and solution; the cell water will increasingly supercool and will eventually freeze *in situ*, usually with lethal consequences.

For the past seven years, our laboratory has been studying various conditions under which IIF occurs or does not occur in several cell types; namely, mouse oocytes and early embryos [3], [4], oocytes of the frog *Xenopus* [5], [6] at various stages of development, V79 Chinese hamster tissue culture cells, and the yeast *Saccharomyces cerevisiae* [7]. Based on physical chemical equations and knowledge of certain parameters such as L_p_ and its temperature coefficient or activation energy, E_a_, one can compute the likelihood of IIF as a function of temperature and cooling rate [2]. By comparing that likelihood with experimental observations on mouse oocytes, mouse embryos and on yeast cells, we have found the agreement to be excellent.

The current paper deals with the water permeability of COS-7 fibroblasts. This tissue cell line is widely used in cell biology as a convenient protein expression system when specific proteins are to be studied. The average diameter of COS-7 cells (18.51 µm) is about two to three times that of most other mammalian cells, making its volume 8 to 27 times higher. Individual cell diameters range from 9 to 33 µm; hence, individual (isotonic) cell volumes of COS-7 cell can differ by as much as 44-fold. These facts made it of interest to determine experimentally the relation between IIF in these cells and cooling rate, to determine the temperature at which IIF occurs, and to compare the observed relation between cooling rate and IIF with the computed relation. As mentioned, that computation requires knowledge of L_p_ and its activation energy, E_a_. Determining these parameters was the purpose of the present study.

The standard way to determine L_p_ is to transfer cells from an isotonic solution to a hypotonic or hypertonic solution of an impermeant solute and determine the rate at which the cell swells or shrinks, respectively. Usually, the L_p_ that is calculated from the rate of swelling has about the same numerical value as the L_p_ calculated from the rate of shrinkage. But occasionally, the two values for L_p_ differ; i.e., the resistance of the membrane to inflow of water differs from its resistance to outflow. Such a difference is referred to as rectification. We have found that COS-7 cells exhibit extremely large apparent rectification; i.e., the value of L_p_ for the influx of water is as much as 18-fold larger than the value for the outflow of water.

## Materials and Methods

### Isotonic and anisotonic test solutions

Test solutions were made from Tyrode's Buffered Saline (TBS). It has a measured osmolality of 0.308, a value that we define as isotonic. Hypotonic solutions were made by diluting TBS with HPLC grade water; hypertonic solutions were made by adding sucrose to the TBS (all the above were from Sigma Aldrich). All test solutions were prepared with a 20% higher or lower osmolality than the desired final concentration to compensate for two subsequent dilutions with 10% volumes of isotonic TBS. One dilution was used for adding 10 µM of the live-dead dye calcein AM (Invitrogen, Carlsbad, CA); the second was for the subsequent addition of the COS-7 cell suspension. The osmolalities were measured with a Vapro Osmometer 5520 (Wescor Inc., Logan, UT). The final milliosmolalities of the TBS test solutions (3–5 samples each), after the 20% volume additions were: 157±2, 242±2, 308±2, 602±6, and 986±3 (Mean ± S.E.).

### Cell culture

COS-7 cells (African Green Monkey kidney fibroblast) were obtained from ATCC (Manassas, VA). They were cultured in Dulbecco's Modified Eagle Medium (DMEM), supplemented with 10% FBS (Invitrogen), in a 5% CO_2_-air atmosphere, at 37°C. After reaching confluency in 3–4 days, the cells were harvested by rinsing in Dulbecco's Phosphate Buffered Saline (Sigma Aldrich). We then dissociated the adherent layer with CellStripper (Mediatech, CA, USA), followed by a quench in DMEM. Harvested cells were pelleted in a 15 ml centrifuge tube (Falcon) at 60× g for 10 min. The pellet was resuspended in TBS. The centrifugation and resuspension was repeated once and the pellet was finally resuspended in a total volume of 180 µl of TBS supplemented with 14.5 mM D-glucose and 0.3% bovine serum albumin (BSA) (Sigma Aldrich). We then added 20 µl of TBS with 10 µM calcein AM to the cell suspension to yield 200 µl of medium and a 1 µM final concentration of calcein AM. The cells were left in this isotonic solution, in the dark at room temperature (RT, 22°C) for at least 30 min before starting the first experiment. The cell suspensions were continuously kept at 22°C until mixing with the test solutions.

### Osmotically induced volume change of individual cells after 5 min at 22°C (RT)

Cell volumes were measured after equilibration in media that had osmolalities below, above, and at isotonicity, and plotted vs. the reciprocal of the osmolality of the medium. This result is referred to as a Boyle-van't Hoff plot (BVH). When the result is linear, the cells are said to behave as ideal osmometers. To obtain a BVH for COS-7 cells, 40 ml of cell suspension (in isotonic TBS+calcein AM) was added to 160 µl of test TBS in the wells of a 96 well plate. The osmolalities of the test solution after the addition of the cells ranged from 157–986 mOsm/kg. After 5 min, the cells had sunk to the bottom of the wells and they were then photographed with a Zeiss Axioscope microscope at 400× over the ensuing 2 min, using both bright field and fluorescence microscopy. The fluorescent excitation maximum for calcein AM is 488 nm. Emission was collected through a 520–700 nm wide pass FITC filter. The diameters of the cells were measured with Image J 1.42Q software (NIH) and their volumes were calculated (Microsoft Excel software) assuming them to be spheres. The 5-min time period between mixing of cells and the measurement of diameters is important. We have shown that with additional time, swollen cells begin to return to their isotonic volume because of volume regulation [8].

### The determination of L_p_ of individual cells by following the dynamics of their volume change in anisotonic solutions at 22°C

**Figure 1:**
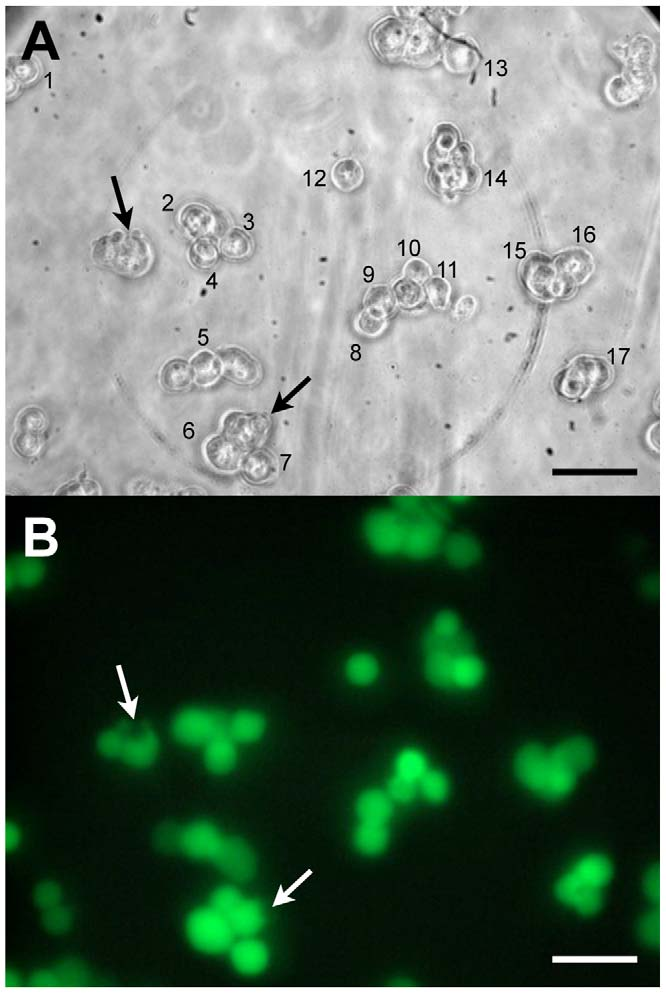
COS-7 cells suspended in the MicroCell chamber at 22°C. A) First image from a series of images initiated 21 s after cells had been exposed to hypotonic (157 mOsm/kg) TBS. The numbers indicate individually identified cells selected for analysis. The changes in their diameters were recorded until well after the cells had reached their maximal volume. Arrows point to two cells that show membrane damage in the form of a bleb of leaking cytosol. B) Fluorescence image of the same group of cells taken immediately after the last image of the bright light image series, about 5 min after A) was taken. Viable cells display green calcein AM fluorescence. Although the two damaged cells still display fluorescence, such cells were excluded from the analysis. Scale bar = 50 µm.

When cells are placed in hypotonic or hypertonic solutions of solutes to which the cells are impermeable, they swell or shrink, respectively, and the rate of that swelling or shrinkage is a measure of the permeability of the cell to water; i.e., the hydraulic conductivity, L_p_. To make these measurements for COS-7 cells, the cell suspension was mixed 1∶4 with TBS solution that had been diluted with water, to yield a final osmolality of 157 mOsm/kg or they were mixed with TBS that had been supplemented with sucrose to yield a final osmolality of 602 mOsm/kg. These are approximately half and twice isotonic. These mixed solutions were immediately loaded into the 50 µm deep chambers of MicroCell slides (Conception Technologies, San Diego, CA) and then transferred to the stage of the microscope for photographing at frequent intervals, an example of the first image of such an imaging series is shown in Fig. 1a. To minimize the amount of cell swelling or shrinkage that occurred before obtaining the first microscope image, the MicroCell slides were kept near 0°C, on an aluminum block immersed in ice, all the test solutions were kept on ice too. First, 16 µl of anisotonic TBS test solution was pipetted into a 0.2 ml plastic tube. Next, 4 µl of cell suspension with calcein AM (held at 22°C) was added, and the resulting suspension was mixed for 2–4 s. The mixed solution warmed to about 6°C. Immediately after the mixing, 4 µl of this cell/test solution mixture was allowed to flow into the chamber of the MicroCell slide. The slide was on ice so the suspension again cooled towards 0°C. Finally, the slide was quickly transferred onto the microscope stage, which was at room temperature. We estimate that the slide converged on that temperature in 10–20 s. This time was used to adjust the focus and to locate a microscope field that displayed at least twenty cells. For each experiment, the time from the beginning of the mixing of the cell suspension with the test solution until the recording of the first image was determined to be 30–40 s. Images were recorded at 10 s intervals for 5 min. This procedure was repeated 2–4 times per test solution. After the last image of a sequence was obtained, the illumination mode of the microscope was set to fluorescence and a fluorescence image was taken, an example of such a fluorescence image is shown in Fig. 1b. The fluorescence images served to determine which cells were dead and therefore needed to be excluded from analysis. To exclude any bias from the added calcein AM, we recorded two control runs under exactly the same conditions, but without calcein AM.

### Dynamics of volume change of individual cells in response to anisotonic solutions at 10°C and 0°C

The exposure of the cells to hypotonic and hypertonic TBS (157 and 602 mOsm/kg), at 10 or 0°C, and the image recording, were performed in a BCS 196 cryostage (Linkam Scientific Instruments, Waterfield, UK) controlled with Pax-it control and capture software (v. 6.1, by Midwest Information Services, Franklin Park, IL). The cryostage was attached to a Zeiss bright-field photomicroscope equipped with a Paxcam digital CCD camera (800××600 resolution) and an Olympus 20× long working distance microscope objective. Prior to its being loaded with cells and inserted into the BCS 196 cryostage that had been precooled to 10 or 0°C, the metal sample holder was kept on an ice-cooled aluminum block. A 12 mm round glass cover slip (Ted Pella No. 26023) was inserted in the sample holder. To prevent squeezing of the cells, a 40 µm thick, round plastic spacer with 10 mm outer and 5 mm inner diameter was placed on this cover slip. Preparation, storage and mixing of the solutions were carried out as described for the RT experiments, except that no calcein AM was added because the Zeiss photomicroscope lacked fluorescence capabilities. After mixing the TBS test solution (cold) with the cell suspension (22°C), 4 µl of this solution was pipetted on the center of the cover slip. A second 12 mm round glass cover slip (precooled) was then placed with vacuum tweezers on this droplet, thereby enclosing the cells in a 40 µm deep cylinder of the test aqueous solution.

The sample holder was taken from the aluminum block and quickly placed into the cryostage, which also had been precooled and kept at 0 or 10°C. The focus was adjusted and an area that displayed at least twenty cells was located. The image sequences were recorded at 10 s intervals for 5 min. The intervals were then increased to 30 s and imaging continued for another 10 to 20 min. This procedure was repeated 2–4 times per test solution. For control purposes, we determined the cell volumes in isotonic TBS at 10 and 0°C. This was done by loading the imaging chamber with cells suspended in isotonic TBS, inserting the sample holder in the cryostage (held at 0 or 10°C), waiting for 5 min, and then imaging cells throughout the whole chamber over the ensuing 2 min. The time from the beginning of the mixing of the cell suspension with anisotonic TBS until the recording of the first image of the image sequence was documented for each experiment. That time was 25–40 s. Because this Zeiss microscope was not equipped for fluorescence imaging, we performed separate test runs with cells that had been preincubated with calcein AM and were subsequently treated exactly the same way as in the swell/shrink experiments at 10 and 0°C. After the imaging sequence in the Zeiss microscope, the sample holder and the imaging chamber were transferred to a fluorescence microscope (Zeiss WL Standard) equipped with FITC filters and the cells throughout the whole chamber were examined for their calcein AM fluorescence.

### Statistics

Values are arithmetic means. Plus/minus values are standard deviations of the mean (standard errors). Tests of statistical significance were carried out by a two-tailed Students t test. All calculations were performed with Microsoft Excel software.

### Determination of the non-osmotic volume (≈volume of solids, V_b_), the hydraulic conductivity, L_p_, and its activation energy, E_a_

A Boyle-van't Hoff relationship was established by plotting the mean of the individual cell volumes (calculated from the recorded diameters, assuming the cells to be spherical), determined in each of the five different osmolality solutions, against the reciprocal of the osmolality of that respective solution; i.e.,
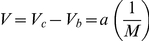
or
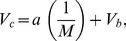
(1)where *V* is the absolute volume of cell water, *V_c_* is the cell volume, *a* is the slope, ***M*** is the osmolality of the test solution, and *V_b_* is the absolute volume of the cell when 1/***M*** is extrapolated to zero (i.e., ***M*** becomes infinite). It is the volume of solids and nonosmotic water in the cell.

Values for the water permeability coefficient, or hydraulic conductivity, L_p_ (µm^3^/µm^2/^min/atm), were determined from the slopes of the plots of change in cell volume vs. time, in either the hypotonic solution (157 mOsm/kg) or in the hypertonic TBS (602 mOsm/kg). The volume of each individual cell at each point in time was calculated for the two tested osmolalities and for all three different temperatures. These individual values were then averaged for all the analyzed cells of an experiment (from 48 to 63 cells), generating six averaged swell/shrink curves. Average L_p_'s for the six combinations of osmolality and temperature were then calculated using the following equation [9], [10], which is the integrated form of the standard differential equation for cell shrinkage or swelling in anisotonic solutions:
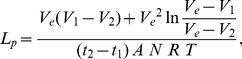
(2)where *V_e_* is the volume of water in the cell after the cell has reached its equilibrium volume, *V_1_* is the cell water volume at time *t_1_*, *V_2_* is the cell water volume at time *t_2_* (all in µm^3^), *A* is the surface area of the cell (µm^2^), *N* is the number of osmoles of solute in the cell, *R* is the universal gas constant (µm^3^.atm/mole.deg), and *T* is the temperature in Kelvin. The several cell water volumes are obtained by subtracting the non-osmotic volume from the cell volume; i.e., *V_c_−V_b_*. *A* and *N* are assumed constant. The computations of L_p_ by Eq (2) were made using values of pairs of volumes *V_1_* and *V_2_* corresponding to 12 to 15 pairs of time intervals from the dynamic part of each averaged swell/shrink curve, and taking the average. Using this integrated equation has the advantage that mixing time artifacts do not affect the result. L_p_ only depends on the time difference (*t_2_−t_1_*) and not the starting time. A list of the parameters used in our calculations, as well as their units and their numerical values, is given in Table 1.

**Table 1:** Initial values and constants used to calculate L_p_ with Equation (2).

		*Temperature*			
*Symbol*	*Parameter*	*0°C*	*10°C*	*22°C*	*units*
V_c_	Cell volume				[µm^3^]
V_c iso_	Mean volume of isotonic cell	3853	3920	3324	[µm^3^]
V_b_	Volume of non-osmotic materials in cells	1116	1135	963	[µm^3^]
V_iso_	Volume of water in the isotonic cell	2737	2785	2361	[µm^3^]
A	Surface area of the cell	1188	1202	1021	[µm^2^]
R	Gas constant	8.21××10^13^	8.21××10^13^	8.21×10^13^	[µm^3^.atm/mole.K]
R′	Gas constant	1.987	1.987	1.987	[cal/mole.K]
*M* ***_iso_***	Intracellular osmolality of isotonic cell = osmolality of Tyrodes	0.308	0.308	0.308	[Osm]
N	Osmoles of solute in cell = *M* ***_iso_***×V_iso_ (in liters)	8.43×10^−13^	8.58×10^−13^	7.27×10^−13^	[Osm]

Note: The volumes of cells and cell solids relative to their volumes in an isotonic cell are shown by a lower case V.

The activation energy, E_a_, for L_p_ describes the dependence of the L_p_ of the plasma membrane on the environmental temperature. E_a_ was calculated from the slope of the Arrhenius plot (the natural logarithm of the L_p_ in µm/min/atm, against the reciprocal of the absolute temperature). In the present study, we intended to calculate E_a_ based on the mean L_p_ values obtained for the hypotonic and hypertonic media at the three tested temperatures: 0, 10 and 22°C, according to the equation E_a_ = −R′ * slope. Again, R′ is the gas constant, but here its units are cal/mole.K, and its value is 1.987. The slope is ln(L_p2_−L_p1_)/(1/T_1_−1/T_2_). However, for reasons we shall discuss later, we concluded that the L_p_'s at 22°C are spurious.

## Results

### Osmotic behavior of COS-7 cells after 5 min equilibration (the Boyle-van't Hoff plot)

**Figure 2:**
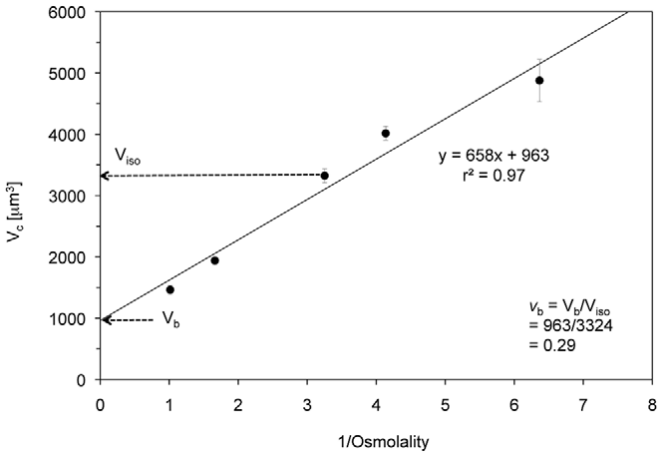
Boyle-van't Hoff plot of COS-7 cells. The BvH plot displays the absolute volume as a function of the reciprocal of the osmolality of the TBS in which the cells were suspended for 5 to 7 min at 22°C. Each point is the mean of 250 to 828 individually analyzed cells. The volumes were calculated from the measured diameters as described in the text. Data were fitted by the method of least squares.

The volumes of COS-7 cells in isotonic media were measured at 0, 10 and 22°C. They were 3853±120 (n = 313), 3920±158 (n = 314), and 3324±61 (n = 828) µm^3^, respectively. The mean isotonic cell volume (V_c iso_) and the volume of water in the isotonic cell (V_iso_) at 22°C were about 15% lower than those measured at 0 and 10°C (Table 1). This difference is significant (p<0.05). To examine whether a Boyle-van't Hoff relationship exists, the cell volumes were determined at 22°C, after the cells had been exposed for 5 minutes to a series of hyper- or hypotonic TBS solutions (range: 157–986 mOsm/kg) and measured during the subsequent 2 min. The volumes of the COS-7 cells exhibited a classic linear Boyle-van't Hoff osmotic response; namely, a monotonic linear relationship with the reciprocal of the osmolality of the medium; the higher the osmolality, the smaller the cells (see Fig. 2). Extrapolation of the linear Boyle-van't Hoff plot to the y-axis generated an average non-osmotic cell volume V_b_ of 963 µm^3^. Normalization, i.e., dividing V_b_ by V_c iso_ (3324 µm^3^), yields a value of 0.29 for the ratio of the volume of cell solids to the volume of the isotonic cell, here referred to as *v*_b_.

### Dynamics of cell volume change in hypertonic TBS (602 mOsm/kg) at 22, 10 and 0°C and the computed L_p_

**Figure 3:**
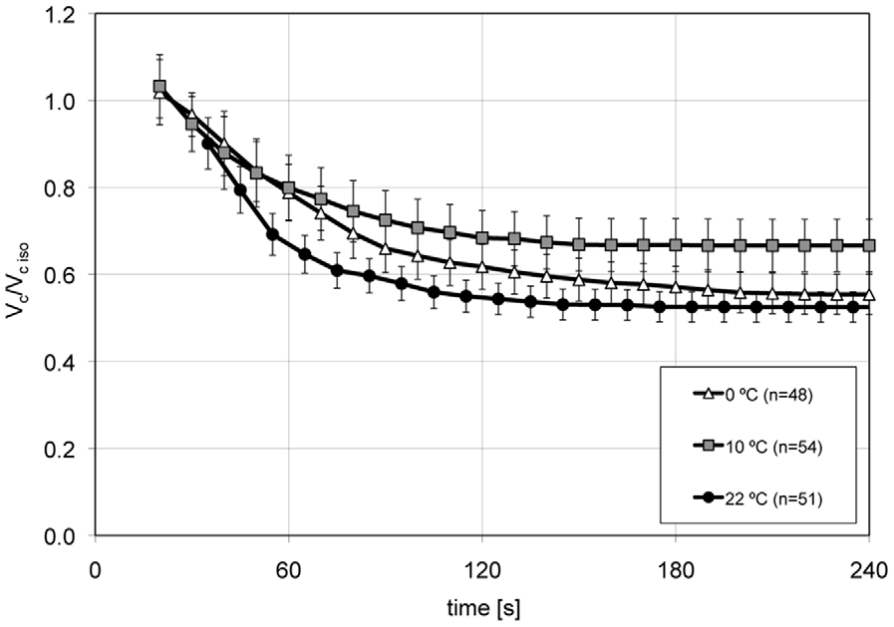
Normalized shrink curves of COS-7 cells in hypertonic (602 mOsm/kg) TBS. The cell volumes were normalized to the mean isotonic volume at each temperature; namely, 3324, 3920, and 3853 µm^3^. The data represent averages and S.E. values of 153 individually measured cells. Cells reached their equilibrium volume faster the higher the temperatures.

We performed kinetic shrink experiments at 0, 10, and 22°C by placing cells in TBS solution made hypertonic (602 mOsm/kg) with sucrose. In the two or three runs per temperature, we analyzed a total of 153 individual cells (n = 51 at 22°C, n = 54 at 10°C and n = 48 at 0°C) by measuring their time-dependent volume changes. Fig. 3 shows the averaged cell volume change at the respective temperatures. The graphs were constructed by first averaging the measured diameters of each individual cell at each recorded point in time. Subsequently the average diameters were converted into cell volumes. As expected, the higher the temperature, the shorter the time until the cells reached equilibrium. At 22°C, they reached their equilibrium volume after 185 s, at 10°C after 230 s and at 0°C after 790 s. The cell volumes at equilibrium were 1762±100 at 22°C, 2626±235 at 10°C and 2199±200 µm^3^ at 0°C. The integrated equation, Eq (2), requires the use of absolute cell volume values (as opposed to relative values) with the units of length being micrometers and the time equal to minutes. These initial values and constants are given in Table 1. The use of this integrated equation to calculate the hydraulic conductivity has the advantage over the standard differential equation method in that it does not require knowing the cell volume at the time of the initial mixing with the test solution and it does not require knowing the initial start time, which is affected by mixing artifacts. The values we obtained for the cell volumes, starting 20–35 s after the initial exposure of the cells to the anisotonic solutions proved sufficient to calculate the L_p_ at 12 to 15 time distinct intervals per temperature curve. Averaging these interval L_p_ values for each temperature resulted in average L_p_'s for COS-7 cells in hypertonic media (602 mOsm/kg TBS) of 0.10, 0.21, and 0.11 µm/min/atm at 22, 10, and 0°C, respectively (see Table 2).

**Table 2:** L_p_ values and their ratios, determined under hyper- and hypotonic conditions at 0, 10 and 22°C.

*Temperature [°C]*	*L_p hyperosm_*	*L_p hypoosm_*	*L_p hyposmo_/L_p hyperosm_*
22	0.10±0.01	1.87±0.10	18.0
10	0.21±0.01	1.73±0.06	8.2
0	0.11±0.01	0.79±0.05	7.5

### Dynamics of cell volume change in hypotonic TBS (157 mOsm/kg) at 22, 10 and 0°C and the computed L_p_

**Figure 4:**
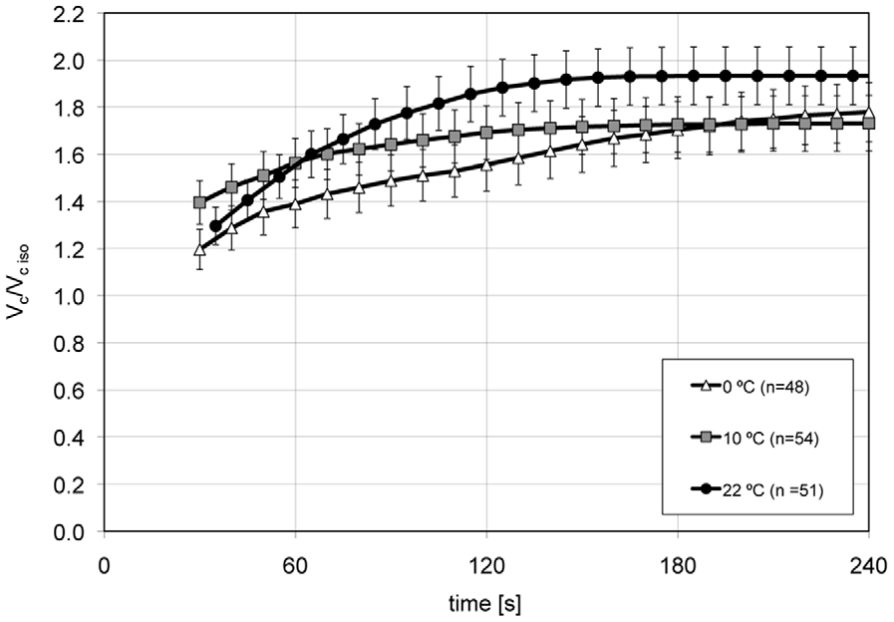
Normalized swell curves of COS-7 cells in hypotonic (157 mOsm/kg) TBS. The data represent averages and S.E. values of 205 individually measured cells. Cells reached their equilibrium volume faster the higher the temperatures. However, these times were significantly longer than in the hyperosomotic situation for all three temperatures tested.

In order to examine if the water permeability is influenced by the flow direction of the water, into or out of the cell, we also performed cell-swelling experiments with hypotonic TBS solution (157 mOsm/kg). These were executed at the same three different temperatures and in the same fashion as the shrink experiments. In two to three runs per temperature, we analyzed a total of 205 individual cells (n = 63 at 22°C, n = 62 at 10°C and n = 80 at 0°C). The resulting graphs are shown in Fig. 4. As in the shrink experiments, the higher the temperature, the less was the time the cells needed to reach their equilibrium volume. The times were 155 s, 180 s, and 610 s at 22, 10 and 0°C, respectively. The cell volumes after these equilibration times were 6548±396, 6782±470, and 7090±462 µm^3^ at 22, 10 and 0°C, respectively. The permeability coefficients for each temperature were calculated using the same procedure as with the hypertonic experiments. Averaging of the interval L_p_ values for each temperature resulted in average L_p_'s for COS-7 cells in hypotonic 157 mOsm/kg TBS of 1.87, 1.73, and 0.79 µm/min/atm at 22, 10 and 0°C, respectively (see Table 2). The differences between the L_p_ values at 0°C and those at 10°C were highly significant (p<0.0005). The average value of L_p_ at 22°C was anomalous, it was essentially the same as the value at 10°C.

### Comparison of water permeabilities (L_p_) in hypo- and hypertonic TBS at 22, 10 and 0°C

**Figure 5:**
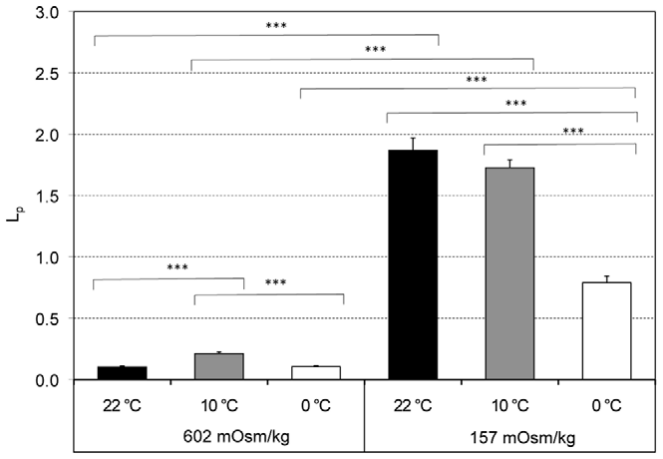
L_p_ values (water permeability) obtained under hyper- and hypotonic conditions. The three bars to the left show the L_p_'s under hypertonic conditions and the three bars to the right under hypotonic conditions. The differences between the two L_p_'s determined at the same temperature are highly significant (p<0.0001). That is true for all three temperatures. These differences strongly indicate rectification of the water flow; that is, the flow rate is different out of the cell than into the cell.

All L_p_ values are displayed in Fig. 5 and in Table 2. Comparing the L_p_ values obtained under hypotonic conditions with their hypertonic counterparts at the same temperature reveals that the values for water influx (hypotonic TBS) are 7 to 18 times higher than the values for water efflux (hypertonic TBS). The differences are highly significant for all three temperature pairs (p<0.001) and increased with rising temperatures, from an L_p hypotonic_/L_p hypertonic_ ratio of 7.5 at 0°C, to 8.7 at 10°C and up to 18.0 at 22°C (Table 1). There was also an effect of temperature causing the L_p_ values in hypotonic conditions at 22°C and at 10°C to be significantly higher than the one at 0°C. In hypertonic conditions, only the L_p_ value at 10°C, but not the one at 22°C was significantly higher than the one at 0°C. The L_p_ value at 22°C was half of that at 10°C and equal to that at 0°C.

### Activation energy (E_a_) of L_p_ in hypo- and hypertonic TBS

**Figure 6:**
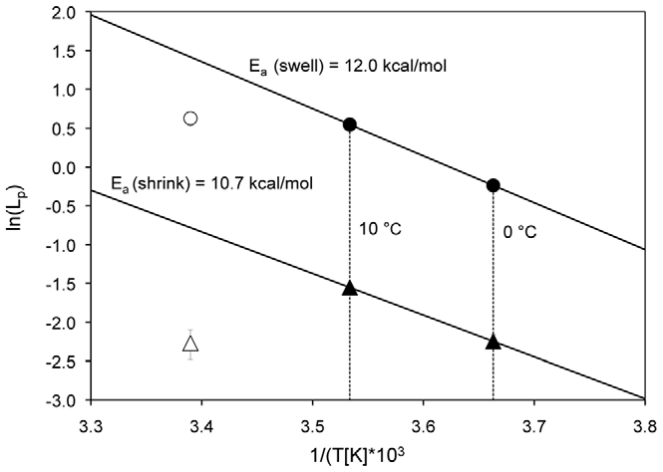
Arrhenius plots for COS-7 cells. The upper line represents the fit for hypotonic conditions (157 mOsm/kg), indicated by circles, and the lower line is the fit for hypertonic conditions (602 mOsm/kg), indicated by triangles, at temperatures of 0 and 10°C. The activation energy, E_a_, of the hypotonic L_p_ values was 12.0 kcal/mol. The value for E_a_ of the hypertonic L_p_ values was 10.7 kcal/mol. The L_p_ values gained at 22°C (open symbols) were not used for the calculation of E_a_, for reasons given in the discussion.

Arrhenius plots were generated for both the hypo- and the hypertonic conditions, by plotting the natural logarithms of the L_p_ values gained at 0 and 10°C, against the reciprocal of the absolute temperature. The activation energy, E_a_, as calculated from the slope of the Arrhenius plot, was 10,700 cal/mol at 602 mOsm/kg and 12,000 cal/mol at 157 mOsm/kg. (see Fig. 6). We did not include the L_p_ values gained at 22°C in the Arrhenius plot for reasons given in the Discussion.

## Discussion

The osmotic and permeability characteristics of cells play an important role in their physiology and they play a central role in determining their responses to the strongly anisotonic conditions occurring during cryobiological preservation. The studies reported here were concerned with determining osmotic parameters and the permeability of COS-7 cells to the osmotically driven inflow and outflow of water. The osmotic questions of concern were do the cells behave as ideal osmometers and what is the fractional volume occupied by cell solids and bound water (*v*_b_)? Answers to both questions can be obtained by measuring the volumes of cells equilibrated in solutions of a range of concentrations of non-permeating solutes, and generating a Boyle van't Hoff (BVH) plot; i.e., a plot of cell volume vs. the reciprocal of the osmolality of the solutions. These are static equilibrium measurements. In contrast, the permeability to water (L_p_) is determined by kinetic measurements in which cells are placed in an anisotonic solution made of non-permeating solutes of known osmolality, and recordings are made of their change in volume with time. In hypertonic solutions, the cells shrink with time as cell water flows out. In hypotonic solutions, the cells swell with time as water flows into the cell. L_p_ is a function of the slope of these fluxes. One can determine E_a_, the activation energy of L_p_, by conducting the shrink/swell experiments at several temperatures. We chose 0, 10 and 22°C.

We studied COS-7 cells for four reasons. One is because this cell line is widely used as a cellular expression system for recombinant proteins and for experiments in molecular biology, biochemistry and cell biology. The second reason is the future possibility of using transfected COS-7 cells that express specific membrane proteins involved in the facilitation of osmotic transport, like aquaporins. Such modified cells would open new ways for detailed studies of cellular mechanisms occurring during freezing and thawing. The third reason is that we wish to compare our so far unpublished observations on the relation between intracellular ice formation in COS-7 and cooling rate with the calculated relationship. To calculate the relationship, we need to know L_p_ and its activation energy. The fourth reason is that the average COS-7 cell has nearly twice the diameter of most mammalian cells, and is about a quarter the diameter of a mouse oocyte. This will allow us to further test the growing impression that the larger a cell, the higher is the temperature at which intracellular ice formation occurs during cooling.

Cells behave as osmometers because of the semipermeable nature of their plasma membrane [11]. The osmotic or chemical potential of water across this membrane depends on both the extracellular osmotic pressure and the intracellular water potential. The former is constant; the latter varies as water leaves or enters the cell. The cellular water volume at a given time can be determined by subtracting the non-osmotic volume, V_b_, from the total cell volume. V_b_ refers to the absolute volume of the intracellular space occupied by lipids, proteins, carbohydrates, and bound water that is tightly hydrogen-bonded to the hydrophilic surfaces of these molecules. It is commonly obtained from the ordinate intercept of a Boyle-van't Hoff plot of the relative cell volume, *v_c_* (the absolute cell volume divided by the volume of the isotonic cell) as a function of the reciprocal of the osmolality of the non-permeating solute. Katkov [12] has recently asserted that this approach is incorrect mathematically and leads to erroneous estimates of *v_b_*. His reason is that when one uses standard formulas to fit a regression line to a plot of normalized cell volume vs. 1/osmolality data, the assumption that the slope and Y-axis intercept of the fitted line are two independent parameters is false. Our approach, however, is not subject to this error because we plot the *absolute* volumes of the cells (V_c_) vs. reciprocal osmolality to obtain the absolute volume of cell solids (V_b_) from the Y-axis intercept of the plot. As shown in Fig. 2, our best estimate of V_b_ is 963 µm^3^.

The potential problem is deciding what value of V_c iso_ should be used in any subsequent normalization. We believe that it should be the mean experimentally measured volume of the COS-7 cells in isotonic Tyrodes (0.308 osmolal) at 22°C. That volume is 3324 µm^3^. This estimate is based on measurements of 828 cells. On that basis, we compute the normalized volume of cell solids (*v*_b_ or *b*) to be 963/3324 µm^3^, or 0.29. One can see that the fitted line in Fig. 2 runs somewhat below the measured value of V_c iso_, and that at the osmolality defined as isotonic (0.308), the corresponding cell volume on the line is 3102 µm^3^. If we normalize all cell volumes to this volume, *v*_b_ (frequently referred to as *b*) becomes 963/3102 or 0.31. However, we think it preferable to normalize to the first isotonic volume (3324 µm^3^) because of the large number of cells measured.

When a cell behaves as an osmometer, the volume of the osmotically available water contained in the cell is inversely related to the osmolality of non-permeable solutes in the external medium [13]. Then, the osmotically active cell volume (V_c iso_−V_b_) indicates the amount of water in isotonic cells that can be lost (unbound water) during the osmotic stress the cell encounters through cryopreservation.

We assessed cell volumes and the changes in cell volumes by microscopic examination of individual cells during exposure to the anisotonic solutions. Although a time-consuming procedure, it had the double advantage of enabling us to evaluate the healthiness and viability of individual cells, as well as giving us the option to examine the osmotic parameters of individual cells. Staining of the cells with the live/dead indicator dye calcein AM and examination in a fluorescent microscope at the end of each experiment revealed survivals of >90% in most experiments and even >95% in some, and permitted us to exclude dead or damaged cells from the analysis. This high survival rate confirmed that our protocol was well adjusted to keep the vast majority of the cells in a viable state during the experiments.

The first parameter we determined was the degree to which the volume of COS-7 cells is related to the reciprocal of the osmolality of non-permeating solutes in which they are suspended. A linear relation in such a BVH plot is indicative of ideal osmotic behavior, and from such plots, as indicated, one can also obtain the non-osmotic volume, V_b_. We found this to be the case for cells suspended for 5–7 minutes in anisotonic TBS solutions (made by the addition of defined amounts of sucrose or of water). The mean diameter of the cells in isotonic media was 18.5 µm and the computed mean cell volume V_c iso_ was 3324 µm^3^ at 22°C. The value of V_b_ at 22°C was 963 µm^3^, which translates to a fractional volume *v*_b_, of 963/3324 or 0.29. This value of *v_b_* or *b* is close to but slightly lower than the values of 0.34 to 0.36 reported for other mammalian fibroblast and epithelial cell lines [14]–[16].

Next, we determined the hydraulic conductivity L_p_, or water permeability of the cells. In this procedure, the cells are mixed with the desired anisotonic solution, loaded into a counting chamber, which in turn is rapidly placed under a microscope, where the cells are photographed at frequent intervals as they shrink or swell. L_p_ is calculated from the rate of shrinkage or swelling [9], [17]–[19]. One potential shortcoming with this procedure is that about 35 s elapsed between mixing the cells with the test solution and the acquisition of the first photographic image. Two steps were taken to minimize the loss of data in that time gap and the consequences of that loss. First, to slow the initial volume changes in that time gap, the solutions and the imaging chamber were kept on ice until the latter was placed on or in the microscope stage. We describe in Materials and Methods the temperature excursions the cells experienced in that interval. With this procedure, we see in Fig. 3 that the relative cell volumes in the first image collected during shrinkage were 1.0 at 10°C and 0°C and 0.9 at 22°C, indicating that little or no shrinkage had occurred at this point. In the swelling experiments, the cell volumes in the first image were 1.2 to 1.4 indicating that some, but not an excessive amount of swelling had already occurred. Second, to calculate L_p_, we used the integrated version of the water permeability equation. It does not require data from the very early portions of the shrink or swell curves but instead uses cell volume/time pairs from randomly chosen intervals of the dynamic part of the curves. This formula has been used previously to calculate the L_p_ of mouse ova [10].

In Fig. 5, we see a striking and highly significant (p<0.001) difference between the L_p_ values calculated for water inflow (hypotonic solutions) and water outflow (hypertonic solutions). At 10°C the L_p_ values differ by a factor of 8 (see Table 2). By analogy with electrical properties, such differences in water permeability between influx and efflux are referred to as *rectification*. It has been much more commonly observed in plant cells and algae [20]–[22] then in mammalian cells. However, rectification was recently reported for transepithelial water flow through confluent mammalian epithelial cells [23], and it has been previously reported in two studies on other mammalian cells. Toupin *et al.* [24] used a Coulter cell sizer to determine the shrinkage and swelling kinetics of human granulocytes in anisotonic solutions. They derived L_p_ values of 2.04 µm/min/atm for exo- and 6.05 for endoosmotic flow at 20°C. Another study, by Muldrew *et al.* [25], also found, by the same Coulter method, that the L_p_ values of V79 Chinese hamster fibroblast cells were higher for endo- than for exoosmotic flow, i.e. 2.9–1.1 µm/min/atm versus 1.3–0.4 µm/min/atm. Although the ratio of endo to exo is 2 or higher, the authors specifically reject calling it rectification, for reasons we find obscure. Rather, they argue that the difference in L_p_ is a consequence of the initial volume of the cells; i.e., the larger the cell, the higher is L_p_. But if so, then the value of L_p_ should continually change as the cell changes volume in both hypertonic and hypotonic solutions. Available evidence does not support that supposition, e.g., [26]. Furthermore in our study, we found that differences of up to 5-fold in the initial isotonic volumes of individual COS-7 cells had no influence on L_p_ (data not shown).

One final example of rectification has been reported in zebrafish embryos by Hagedorn *et al.* [26]. Interestingly, the direction of the rectification there is opposite what we and the other authors cited above have found in mammalian cells; i.e., in zebrafish, the L_p_ for endosmosis is much lower than that for exoosmosis. It is attractive to assign an evolutionary explanation for this. Zebrafish are fresh water animals and their oocytes are shed into freshwater and fertilized there. Consequently, there is a strong osmotic force to drive water into the cells. Due to the fact that the L_p_ for endoosmotic flow is near 0, the rate of that endosmosis is greatly slowed down.

It should be noted that in our experiments the osmolalities of the external media used to determine L_p_ differed by a factor of 3.8 (602 mOsm/157 mOsm) in the water efflux and water influx experiments, respectively. The L_p_ values differed by a factor of 7–18, depending on the temperature. The question is which is responsible for this difference – the direction of water flow or the difference in the external osmolality?

House [27] has discussed this question in detail in his monograph on water transport in cells and tissues. In some cases, the apparent rectification is due to the fact that L_p_ shows a reciprocal relation to the osmolality of the external medium. That is to say, the lower osmolalities used to assess the L_p_ during swelling yield higher values of L_p_ than the high osmolalities used to assess L_p_ during cell shrinkage. Armitage [28] has published an elegant demonstration that the apparent rectification in human platelets is due entirely to this effect of the external osmolality on L_p_. A second class of findings is that the L_p_ is essentially independent of the external osmolality and that differences in its value between swelling and shrinkage represent true rectification. The third case is where the two explanations are experimentally confounded. The fourth and perhaps most common case is where L_p_ is independent of the direction of water flow and independent, or nearly so, of the osmolality of the external solution; i.e., there is no evidence of rectification of any sort.

The third case is the situation in our experiments. Armitage's approach was to subject the platelets to small changes in external osmolality (mostly ranging between 20–40 mOsm/kg). He found that the direction of water flow had no effect on the estimated L_p_, but the final osmolality of the external medium had a major effect. However, his method is not applicable to our procedure. He used a photometric method that allowed instantaneous detection of volume changes in thousands of cells at the time of mixing and with a resolution of fractions of a second. We measured the volumes of individual cells with the first image being taken ∼35 s after mixing cells and medium. In addition, platelets differ significantly from COS-7 cells in many aspects, such as a 40 fold higher surface area to volume ratio, a 100 to 2000-fold lower initial volume, and a three-fold smaller value of *v*_b_.

One problem we have with the external osmolality hypothesis is why should an effect of osmolality on L_p_ be restricted to the external osmolality? Why not an effect from the intracellular osmolality? In our swelling experiments, the external osmolality is fixed at 157 mOsm/kg, but the internal osmolality decreases from 308 mOsm/kg at 0 time to 157 mOsm/kg at equilibrium. In our shrinkage experiments, the external osmolality is fixed at 602 mOsm/kg, but the internal osmolality increases from 308 mOsm/kg at 0 time to 602 mOsm/kg at equilibrium. If such changes in internal osmolality had substantial effects on L_p_, then the later the pairs of times chosen for the calculation of L_p_ by Eq (2), the greater should have been the difference from the L_p_'s calculated from early pairs of times; however, we could not detect any such effect in our data, in fact it is tantamount to saying that a single value of L_p_ appears to hold sway during the entire shrinkage or swelling curve.

**Figure 7:**
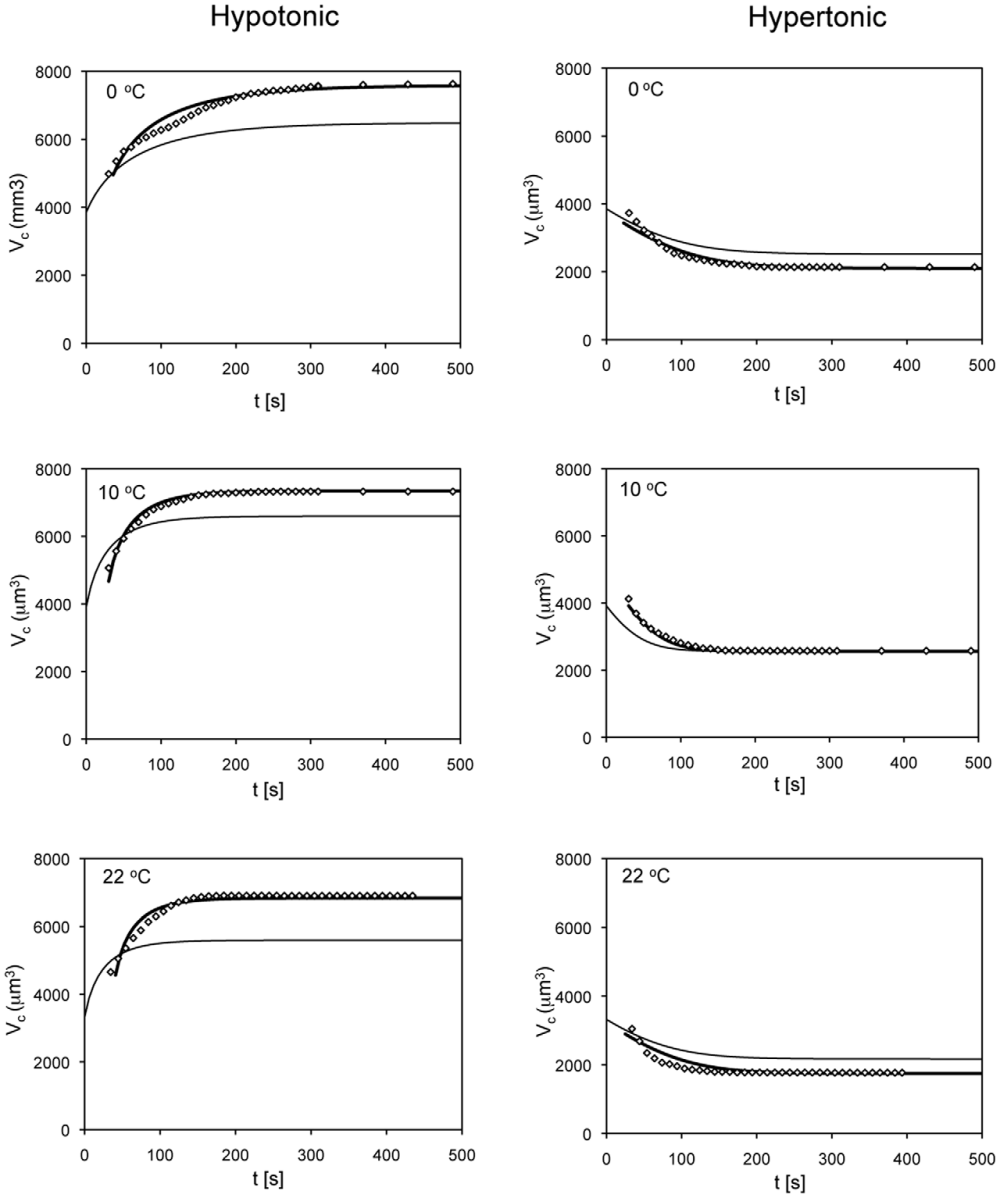
Comparison of the experimental data with computer simulations. Plots of the volumes of COS-7 cells as function of time under hypotonic conditions (left column) and under hypertonic conditions (right column). The diamonds are the experimental values. The light solid line is the simulated curve obtained by calculating the cell volumes as a function of time using the classical differential equation [Eq 3]. The heavy solid line was obtained by sliding the light line in the X and Y directions to obtain the best fit by eye to the experimental points.

There is another way to examine the question of whether a single value of L_p_ operates over the whole range of shrinkage and swelling of the COS-7 cells. First, take the mean L_p_ values obtained from applying the integrated equation (Eq. 2) to pairs of V and t experimental values and incorporate those L_p_'s into the differential equation that describes the kinetics of water loss or water gain. Second, use that equation to compute the entire kinetic curve. And third, compare those simulated curves with the experimental points. These calculations and comparisons are shown in Fig. 7 for each of the six conditions used. In each graph, the points are the experimental values and the thin solid line is the simulated shrinkage or swelling curve. These simulated curves are generated by solutions to the differential equation
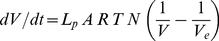
(3)where *V* refers to the absolute volumes of water, and the other symbols have the same units and meaning as in Eq. (2). The values of the L_p_ used in each condition are given in Table 2. We see that the shapes of the simulated curves appear similar to the experimental but are shifted both with respect to time and cell volume. The heavy solid curves are the result of manually shifting the simulated curves with respect to X and Y so as to provide the best fit by eye to the experimental points. In almost every case, the shifted simulated curves coincide almost exactly with the experimental points.

In the case of the time axis, the shift is always to the right and amounts to a mean 30.8 s increase in time. In Fig. 7, as well as in Figs. 3 and 4, the value of 0 on the time axis is the time the cells are first mixed with the test solution. In the simulated curve, that mixing is assumed to be instantaneous and complete. Consequently, the cells are assumed to begin shrinking or swelling instantaneously. In the experiments, a measured 25 to 40 seconds elapsed between the initial mixing and the acquisition of the first photographic image.

In the volume axis there are two differences between the experimental and the simulations. The first is that the volume of the cells at 0 time in the simulation is assumed to be the mean measured value of the isotonic cells at each temperature. In the experimental runs, we had no measurement of the volume of a given cell at 0 time. A second difference is that the simulation curves assume that the final, equilibrium, water volume obeys the Boyle-van't Hoff law, which means that the ratio of the equilibrium water volume to the isotonic water volume (mean = 2626 µm^3^) is equal to the ratio of the isotonic osmolality (0.308) to the osmolality of the test solution (0.157 or 0.602); i.e., 1.96 or 0.512. Or, in absolute terms, the equilibrium volumes of cell water V, after swelling or shrinking are 5147 and 1345 µm^3^. To calculate the equilibrium *cell* volumes, one must add the V_b_ of 963 µm^3^. This leads to calculated equilibrium cell volumes of 6110 and 2308 µm^3^ for the hypotonic and hypertonic cells, respectively. For reasons still not clear, the calculated equilibrium volumes do not coincide with the experimental equilibrium volumes. In the hypotonic measurements, the experimental equilibrium cell volumes are greater than the calculated equilibrium values; in the hypertonic measurements, the situation is reversed. Still, when the simulated curves are appropriately shifted up or down, they coincide almost exactly with the experimental points.

We draw two conclusions from this close correspondence between the shifted simulation curves and the experimental points. The first is that calculating L_p_ from the integrated equation (Eq. 2) is a valid approach; that is, by incorporating pairs of experimental cell volume/time data into the integrated equation, one obtains a value of L_p_ that generates a full swell or shrinkage curve that provides a very close fit to the experimental points. The second conclusion is that a single value of L_p_ suffices to produce this close fit over the entire range of cell volumes during hypertonic shrinkage or hypotonic swelling.

The second important osmotic parameter, the Arrhenius activation energy, E_a_, of L_p_, was first calculated by plotting the natural logarithm of L_p_ obtained at 22, 10 and 0° C against the reciprocal of the absolute temperature (Fig. 6). One set was prepared for the exosmosis data and one set for the endosmotic data. According to Arrhenius' theory, the plot should yield a single straight line, with a slope proportional to the activation energy for the process; namely, the movement of water either into or out of the cell. If we were to base the line on the L_p_ at all three temperatures, it clearly was not a straight line and would yield very low values of E_a_. Consequently, we decided that the data obtained at 22°C were spurious and therefore did not include them in the final determination of E_a_ (see Fig. 6). We believe the apparent L_p_ values at 22°C were abnormally low because they were affected by the counter influence of volume regulation at this temperature. We have shown that COS-7 cells display a marked regulatory volume decrease at 22°C when exposed to a hypotonic TBS solution of 157 mOsm/kg [8]. This effect is not present at 0 and 10°C, indicating that it is an energy requiring process. In other words, at 22°C the passive inflow of water is partially opposed by the regulatory outflow of water, and this results in the measured L_p_ being lower at 22°C than would otherwise be the case. This opposition to water flow does not occur at 10 and 0°C. When we base our estimates of E_a_ on the L_p_ values gained at 0°C and 10°C, the E_a_ for endoosmotic flow amounts to 12.0 kcal/mol and that for exoosmotic flow is 10.7 kcal/mol.

The values of L_p_ and its E_a_ have been considered diagnostic for the presence or absence of aquaporin pores in the plasma membrane [29]. The combination of a high L_p_ (e.g., >1 µm/min/atm) and a low E_a_ (e.g. <6 kcal/mole) is considered to indicate permeation through aquaporins. In contrast, the combination of a low L_p_ (e.g., <0.6 µm/min/atm) and a high E_a_ (e.g., >10 kcal/mole) indicates the absence of pores and the permeation of water by diffusion through the lipid bilayer of the plasma membrane. In this regard, our results for COS-7 cells are paradoxical. In the case of endosmosis, the L_p_'s are very high (1.73 µm/min/atm at 10°C), which supports water permeation through aquaporin pores, but the E_a_ is also very high (12.0 kcal/mole), which opposes the idea of water flow through aquaporin pores. However, in the case of exoosmosis, the low L_p_ values of <0.10 µm/min/atm in combination with an E_a_ value of >10 kcal/mole clearly suggests bilayer diffusion.

Conclusions about the mechanisms of water transport in COS-7 based on E_a_ values must be viewed cautiously. First of all, although we made measurements of L_p_ at three temperatures, we have concluded that the only valid measurements are those made at 10 and 0°C. The values of L_p_ at these two temperatures are based on measurements of the volumes of 142 cells, and, therefore, we believe they are accurate. Adding measurements at one or two temperatures between 10 and 0°C might have increased our comfort level in the value of E_a_, but it would not have told us whether this value can be extrapolated to subzero temperatures. It is the value below 0°C that is especially important to an analysis of the probability of a cell undergoing intracellular ice formation.

To solve the remaining open questions related to osmotic and permeability matters described here, future work with COS-7 cells might use transfected cells expressing different aquaporins and might examine the influence of cytoskeleton associated proteins, like integrins and cadherins, that may be candidates for transferring osmolality related signals to the membrane [30].

In conclusion, we describe here for the first time the osmotic parameters such as the non-osmotic volume, the water permeability, and the activation energy of COS-7 cells. The most prominent finding of this study is the dependence of the water permeability coefficient L_p_ on the direction of the water flow in or out of the cell, a phenomenon known as rectification. Rectification has only rarely been documented in mammalian cells. The activation energies for both endo- and exoosmotic flow were high enough in both cases to argue against the existence of aquaporins or other water channel proteins in COS-7 cells. However, the high L_p_ values in endosmosis argue for the existence of aquaporins. The presence of parallel pathways for the water movement in and out of the cell is supported by the effects of regulatory volume decrease under hypoosmotic conditions, which we believe influences the apparent water permeability at 22°C. A paper on this matter is in preparation.
